# Large-scale genetic investigation of nematode diversity and their phylogenetic patterns in New Zealand's marine animals

**DOI:** 10.1017/S003118202200138X

**Published:** 2022-11

**Authors:** Jerusha Bennett, Robert Poulin, Bronwen Presswell

**Affiliations:** Zoology Department, University of Otago, Dunedin, New Zealand

**Keywords:** Acuariidae, Anisakidae, biodiversity, Nematoda, parasite, phylogenetics, Spirurina

## Abstract

Nematodes constitute one of the most speciose metazoan groups on earth, and a significant proportion of them have parasitic life styles. Zooparasitic nematodes have zoonotic, commercial and ecological significance within natural systems. Due to their generally small size and hidden nature within their hosts, and the fact that species discrimination using traditional morphological characteristics is often challenging, their biodiversity is not well known, especially within marine ecosystems. For instance, the majority of New Zealand's marine animals have never been the subject of nematode studies, and many currently known nematodes in New Zealand await confirmation of their species identity with modern taxonomic techniques. In this study, we present the results of an extensive biodiversity survey and phylogenetic analyses of parasitic nematodes infecting New Zealand's marine animals. We used genetic data to differentiate nematodes to the lowest taxonomic level possible and present phylogenies of the dominant clades to illustrate their genetic diversity in New Zealand. Our findings reveal a high diversity of parasitic nematodes (23 taxa) infecting New Zealand's marine animals (62 of 94 free-living animal species investigated). The novel data collected here provide a solid baseline for future assessments of change in diversity and distribution of parasitic nematodes.

## Graphical abstract

**Figure:**
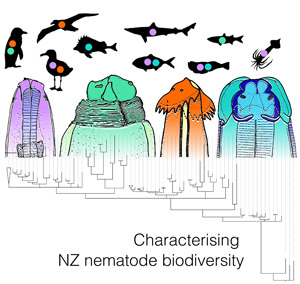

## Introduction

Nematodes are extraordinarily ubiquitous, abundant and diverse. There are more than 40 000 species currently known, thus they represent one of the most speciose metazoan groups on earth (Zhang, 2013). The rate of descriptions is slowly increasing with about 400 new species descriptions per year (Hodda, 2022*a*). However, estimates suggest there are somewhere between 500 000 and 5 000 000 nematode species in total, which is 8–100 times more than that of the current known total (May, 1988; Hugot *et al*., 2001; Hodda, 2022*b*). These estimates and the variability between them make it clear that although considerable effort has been directed towards understanding nematode biodiversity within natural systems, much is left to be discovered. Addressing this deficiency is increasingly important now more than ever, as biodiversity loss is considered an urgent conservation priority across the globe (Butchart *et al*., 2010).

Anderson (2000) estimated that approximately 33% of nematodes currently known are parasitic in vertebrate definitive hosts. Aside from this large contribution to nematode species richness, parasitic nematodes are well recognized for their zoonotic, economic and ecological importance within natural systems, especially in the marine environment. Various marine nematodes are zoonotic; humans who consume undercooked infected flesh can develop mild-to-severe allergic reactions, or acquire accidental infections, either of which can lead to death in severe cases (Audicana *et al*., 2002; Mattiucci *et al*., 2013). In their non-human hosts, nematodes can reduce the fecundity, growth and overall health of their host, also sometimes leading to death (e.g. Wiese *et al*., 1977; Vanstreels *et al*., 2018). At a larger scale, such pathological infections may potentially cause secondary extinctions, especially if the host taxa are threatened. Although nematodes are not typically cited as the primary cause of mortality events, they can be associated as a contributing factor (e.g. Abollo *et al*., 2001*a*). For fish hosts that are commercially important, nematode infections can make fish undesirable for human consumption, reducing the marketability of fillets, and consequently posing a potential economic risk for some fisheries (Abollo *et al*., 2001*b*; EFSA, 2010). Contrary to these negative impacts, parasitic nematodes also play major roles within food webs and perform ecological functions within healthy natural systems (Singleton and McCallum, 1990; Timi and Poulin, 2020). They can be used as biological tags for biomonitoring of host species (e.g. Melendy *et al*., 2005; Poulin and Kamiya, 2015), as indicators of ecosystem health or environmental change (Zarlenga *et al*., 2014) and, due to their high diversity, can be used as models for resolving evolutionary hypotheses regarding species radiation events (e.g. Mattiucci and Nascetti, 2008). Clearly, a wealth of knowledge concerning the structure and functioning of natural systems can be gained from investigating parasitic nematodes within marine ecosystems. Despite this however, they, along with other parasite taxa are often underrepresented or ignored completely in studies of biodiversity and ecology (Lafferty *et al*., 2008).

There are a few reasons why parasitic nematodes are underrepresented in studies of biodiversity. First, nematodes are usually small, making them seemingly insignificant, and live within their host, thereby going undetected. Second, nematodes are often more difficult to identify using traditional morphological features than other metazoan groups, as many exist as cryptic species complexes which lack distinguishable morphological characters between life stages, and between closely related species (Nadler and Pérez-Ponce de León, 2011). This makes it difficult to identify nematodes to low taxonomic levels for non-experts and sometimes even for experts. Lastly, the taxonomy of phylum Nematoda continues to change and evolve at varying taxonomic levels with the addition of new data and approaches (Hodda, 2021). Currently, inventories of nematode taxa that comprise ‘difficult to delineate taxa’ are incomplete (Mattiucci and Nascetti, 2008). However, molecular discrimination methods are increasingly incorporated in studies that aim to identify nematodes at a genotype, population and species level, especially for those groups that comprise cryptic species; these have led to significant advances in our understanding of nematode evolution and ecology (Shamsi *et al*., 2009*b*; Nadler and Pérez-Ponce de León, 2011; Mattiucci *et al*., 2014; Li *et al*., 2018).

In New Zealand, fewer than 10% of animals expected to host parasitic helminths have any records of infection (Bennett *et al*., 2022*a*). Furthermore, Bennett *et al*. (2022*a*) reported that, of the nematodes recorded infecting New Zealand's marine animals, more than half require further study to confirm their species identity. Limited genetic data are available for most nematode–host associations that are known, hindering our ability to corroborate identifications from existing checklists, which include data from fish species (Hine *et al*., 2000), seabird species (McKenna, 2010, 2018) and marine mammals (Lehnert *et al*., 2019). However, some studies focusing on delineation of nematode groups have included a few New Zealand animals previously (e.g. *Anisakis* as per Mattiucci *et al*., 1997, 2018). The uncertainty of current data for New Zealand nematodes reflects a lack of taxonomic effort, with the sources of current records biased towards purposes such as fisheries management (Bennett *et al*., 2022*a*). Going forward, obtaining genetic data for New Zealand's marine parasitic nematodes from a wide range of host taxa should provide insights into (1) what nematode taxa are present in New Zealand, including unique genotypes, species complexes or species, (2) which free-living animals host nematodes, (3) identification of unknown potentially disease-causing nematodes and (4) the diversity of zoonotic species, as it is not yet understood which genotypes or species are infective to humans (Hochberg and Hamer, 2010). Genetic data would also provide a starting point for future comparison for monitoring changes in diversity or distribution of parasites (Mattiucci and Nascetti, 2008; Faltýnková *et al*., 2017).

With this in mind, the aim of this study is to characterize genetically the biodiversity of parasitic nematodes that infect a range of New Zealand's marine animals. The data presented here result from an extensive parasite biodiversity survey carried out between 2019 and 2021. We took a broad, collaborative and opportunistic surveying approach by utilizing deceased marine animals from around New Zealand from a range of locations and sources. We primarily used molecular data and phylogenetic analysis to characterize the biodiversity of the main nematode clades infecting some of New Zealand's marine animals. It is not our intention to update the current evolutionary hypotheses or taxonomies regarding the nematode groups presented, but rather to use phylogenies to visually illustrate the diversity present in New Zealand waters. We update several unresolved nematode records and provide numerous new geographic and host records. Our findings also serve as a baseline for future studies aiming to understand changes in species composition or distribution of parasites in New Zealand waters.

## Materials and methods

### Host and parasite collection

Between June 2019 and August 2021, a total of 611 individuals of 94 animal species were dissected with the aim of molecularly characterizing the parasitic helminth biodiversity within some of New Zealand's marine animals. Helminths other than nematodes found during this exploration will be reported elsewhere. Marine animals examined for nematodes were obtained from a range of sources and locations around New Zealand. All were provided deceased as by-catch or as a by-product of other research, except for a few inter- or sub-tidal fish species that were collected using hand nets and euthanized under a University of Otago animal use protocol (permit AUP-19-190). All animals were obtained between June 2019 and August 2021; Supplementary material 1 details each host species investigated, with locality data where known. Note that in some cases location data were not provided when information pertained to confidential fishery by-catch pelagic seabirds; such records are reported as caught within New Zealand's exclusive economic zone (EEZ). The host taxa investigated include mostly vertebrates, including 39 seabirds, 40 teleost fish, 10 chondrichthyan and 1 marine mammal species. We also include data from 4 cephalopod species as they are known intermediate/paratenic hosts for some marine nematodes in New Zealand (Smith *et al*., 1981). Other invertebrate species were also dissected during this survey, only 1 of which hosted parasitic nematodes; those results are presented in a checklist of parasites infecting New Zealand's marine invertebrates (Bennett *et al*., 2022*b*).

Marine animals were defrosted if frozen or dissected fresh. Organs within hosts examined for nematodes differed depending on host taxa. For teleost fish and chondrichthyan hosts, muscle tissue, gastrointestinal tract and internal organs were removed and dissected. For seabirds, gastrointestinal tracts, and in some cases lungs, were removed and dissected. For marine mammals, gastrointestinal tracts or fecal samples were investigated. Lastly, for cephalopods, internal cavities were dissected and investigated.

### Molecular data

Representatives of each nematode species, when numbers and condition allowed, were chosen for DNA sequencing. Genomic DNA was extracted using the DNeasy^®^ Blood and Tissue kit (Qiagen, Hilden, Germany) according to the manufacturer's protocol. First, a partial sequence of the small subunit rDNA 18S gene was amplified, primarily to identify nematodes to the lowest taxonomic level possible. Next, cytochrome c oxidase subunit 1 (*cox1*) gene was targeted for representatives from the genus *Anisakis*, internal transcribed spacers 1 (ITS1) and 2 (ITS2) genes were targeted for representatives of the genera *Contracaecum* and *Hysterothylacium*, and the large subunit rDNA 28S gene for species belonging to Acuariidae, to allow comparison between closely related species (choice of markers was based on previous studies: Cross *et al*., 2007; Shamsi *et al*., 2009*b*; Costa *et al*., 2018; Mutafchiev *et al*., 2020, respectively). Polymerase chain reaction (PCR) protocols for the 18S gene included primers Nem18SF and Nem18SR (Wood *et al*., 2013) and conditions consisting of 94°C for 5 min, 35 cycles of 94°C for 30 s, 54°C for 30 s and 72°C for 1 min, and 72°C for 10 min. PCR protocols for *cox1* included a primer mix of LCO1490 and HCO (Folmer *et al*., 1994) and conditions followed that of Prosser *et al*. (2013). PCR protocols for ITS1 and ITS2 included primers SS1 N13r and SS2 NC2 (Shamsi and Suthar, 2016), respectively, under conditions of 94°C for 5 min, 30 cycles of 94°C for 30 s, 55°C for 30 s and 72°C for 30 s, and 72°C for 5 min. PCR protocols for 28S included primers T16 and T30 (Harper and Saunders, 2001) under conditions of 94°C for 5 min, 30 cycles of 94°C for 30 s, 45°C for 30 s and 72°C for 2 min, and 72°C for 7 min. PCR products were cleaned using EXOSAP™ Express PCR Product Cleanup Reagent (USB Corporation, Cleveland, OH, USA) following the manufacturer's instructions. Sanger sequencing by capillary electrophoresis was performed by the Genetic Analysis Service, Department of Anatomy, University of Otago (Dunedin, New Zealand), Macrogen Incorporated (Seoul, Republic of Korea) or by Massey Genome Services, School of Fundamental Science, Massey University (Palmerston North, New Zealand).

Sequences were imported into Geneious Prime^®^ v1.2, trimmed using the trim function with default parameters and manually edited for incorrect and ambiguous bases. An alignment was created for each of the main nematode groups recovered, together with sequences of close relatives downloaded from GenBank following BLASTn searches. The alignments were as follows: Spirurina (18S), Acuariidae (18S), *Hysterothylacium* (ITS1 and ITS2), *Anisakis* (*cox*1), *Contracaecum* (ITS1 and ITS2) (Chromadorea: Rhabditida) and Capillariidae (18S) (Enoplea: Trichinellida). For the higher level Spirurina 18S alignment, NCBI searches were performed and a few representatives per suborder were selected manually. Although partial 28S was obtained for some acuariids, no phylogeny is presented; however, newly produced 28S acuariid sequences are listed in Supplementary material 2. One of each unique genotype produced here was selected for comparison in alignments, although all newly generated sequences are submitted to GenBank for future comparison (see Supplementary material 2). When required, the program Gblocks v0.91.1 (Castresana, 2000; Talavera and Castresana, 2007; Lemoine *et al*., 2019) was used to refine nuclear gene alignments, removing poorly aligned regions. In total, 6 alignments and corresponding phylogenetic inferences were performed. Ingroups, outgroups and GenBank accession numbers for downloaded sequences are provided in each figure or figure captions. The program jModelTest v2.1.6 (Guindon and Gascuel, 2003; Darriba *et al*., 2012) was used to estimate the model of evolution for each alignment, restricted to 3 substitution models for compatible use with MrBayes. Models selected were as follows: GTR + I + G for Spirurina 18S alignment, HYK + I + G for Acuariidae 18S alignment, HYK + I + G for *Hysterothylacium* ITS1 and ITS2 concatenated alignment, GTR + I + G for *Anisakis cox*1 alignment, GTR + G for *Contracaecum* ITS1 and ITS2 concatenated alignment and HYK + I + G for Capillariidae 18S alignment. Bayesian inference was conducted for each alignment in MrBayes version 3.2.7a (Huelsenbeck and Ronquist, 2001) using the online interface: Cyberinfrastructure for Phylogenetic Research (CIPRES) Science Gateway (Miller *et al*., 2010). Analyses performed had random starting trees for 2 runs (each with 1 cold and 3 heated chains), employing a Markov Chain Monte Carlo approach for sampling the joint posterior probability distribution across 10 000 000 generations at a heating chain temperature of 0.02°C. The first 25% of samples were discarded as burnin.

After each analysis, mixing and convergence estimates were evaluated through CIPRES output files and Tracer v1.6.0 (Rambaut *et al*., 2018) to ensure appropriateness of each estimated phylogeny. Resulting trees were summarized in a 50% majority-rule consensus tree with clade credibility support values (Bayesian posterior probability, BPP) and branch length information. Trees were visualized in FigTree v1.4.4 (http://tree.bio.ed.ac.uk/software/figtree/) and edited in Inkscape v1.1 (https://inkscape.org). BPP higher than 0.8 was considered moderately supported, and greater than 0.95 was considered strong support for nodal positions. Uncorrected pairwise genetic distances were also estimated in MEGA v10 (Stecher *et al*., 2020).

### Morphological data

Morphological data were gathered from representative nematode specimens to allow identification to the lowest taxonomic level possible. Nematodes were cleared with lactophenol as temporary mounts for light microscopy. Morphological identification was facilitated with the use of ImageJ software (Wayne Rasband, NIH, USA) from photographs obtained on an Olympus BX51 compound microscope mounted with DP25 camera attachment. Parasites were identified to the lowest taxon possible using the morphological keys of Anderson *et al*. (2009) and Gibbons (2010) and original species descriptions.

## Results

**Fig. 1.**
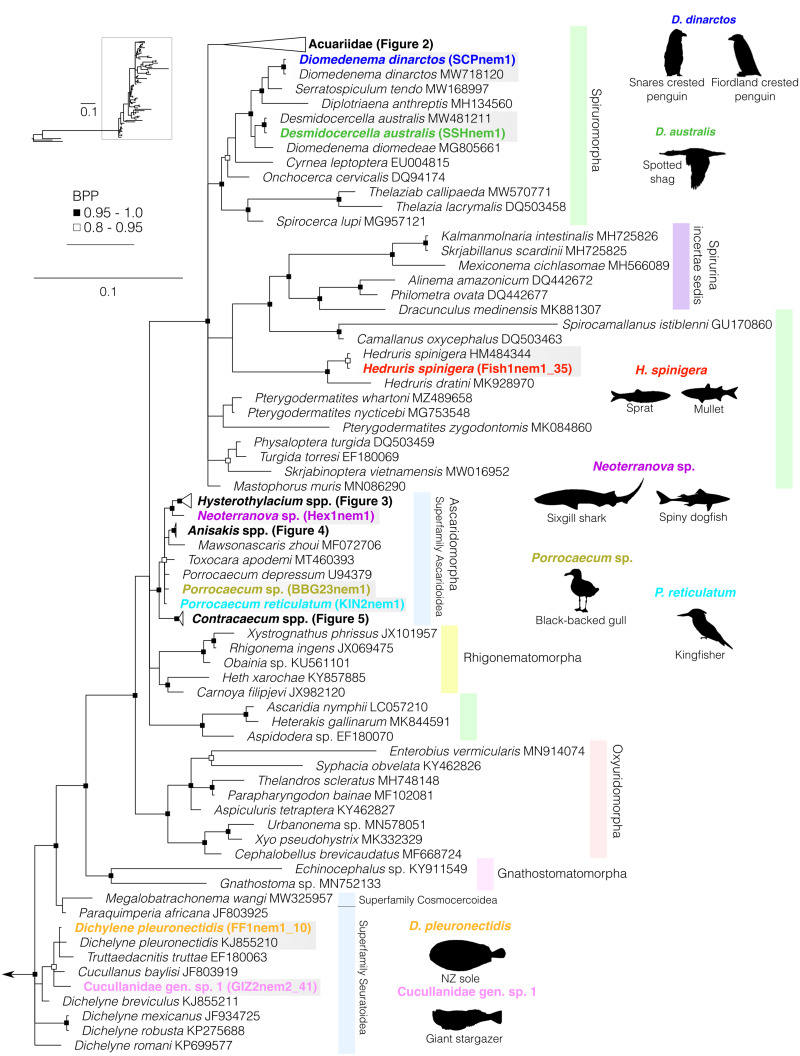
Bayesian phylogenetic inference of nematodes from suborder Spirurina inferred from partial 18S sequence data. Black silhouettes represent definitive hosts. Infraorders or lower taxonomic levels of interest are depicted by shaded boxes to the right of the phylogeny. Coloured sequences represent species recovered in this study from New Zealand's marine animals. BPP denoted by black and black-outlined white squares. Scale represents substitution per base. Outgroups include representatives of suborder Tylenchina, family Aphelenchoididae (MH844706, JQ957895 and JQ975889).

**Fig. 2.**
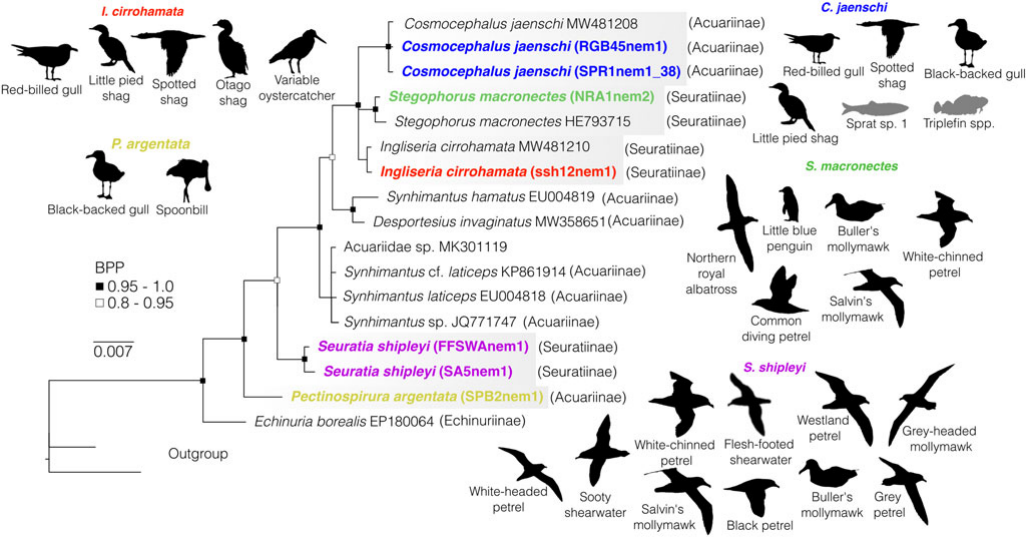
Bayesian phylogenetic inference of nematodes from family Acuariidae inferred from partial 18S sequence data. Black silhouettes represent definitive hosts and grey silhouettes represent intermediate hosts. BPP denoted by black and black-outlined white squares. Scale represents substitution per base. Each colour represents a species recovered in this survey from New Zealand's marine animals. Subfamilies are reported in brackets. Outgroups include representatives of family Desmidocercidae for 18S data (MW481211 and MW718120).

**Fig. 3.**
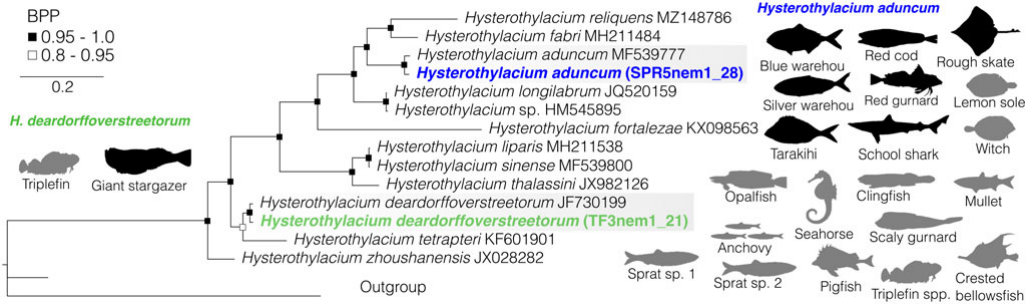
Bayesian phylogenetic inference of nematodes of the genus *Hysterothylacium* (family Raphidascarididae) inferred from ITS1 and ITS2 data. Black silhouettes represent definitive hosts and grey silhouettes represent intermediate hosts. Coloured sequences represent species recovered in this study from New Zealand's marine animals. BPP denoted by black and black-outlined white squares. Scale represents substitution per base. Outgroups include representatives of *Contracaecum* (AJ250415-6 and MW481320).

**Fig. 4.**
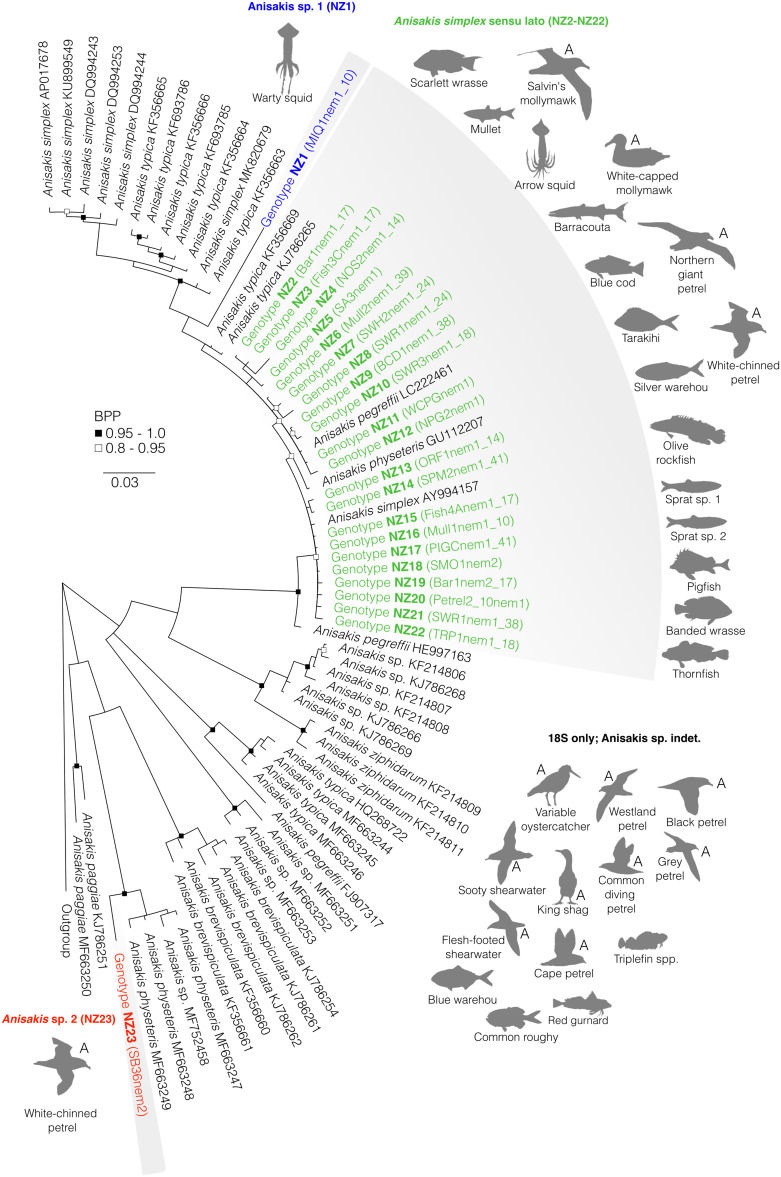
Bayesian phylogenetic inference of nematodes of the genus *Anisakis* (family Anisakidae) inferred from *cox*1 data. Black silhouettes with ‘A’ next to them represent accidental hosts and grey silhouettes represent intermediate hosts. BPP denoted by black and black-outlined white squares. Shaded areas represent species clades; coloured sequences represent those recovered in this survey from New Zealand's marine animals. Scale represents substitution per base. Outgroup includes a representative of *Contracaecum* (MW133972).

**Fig. 5.**
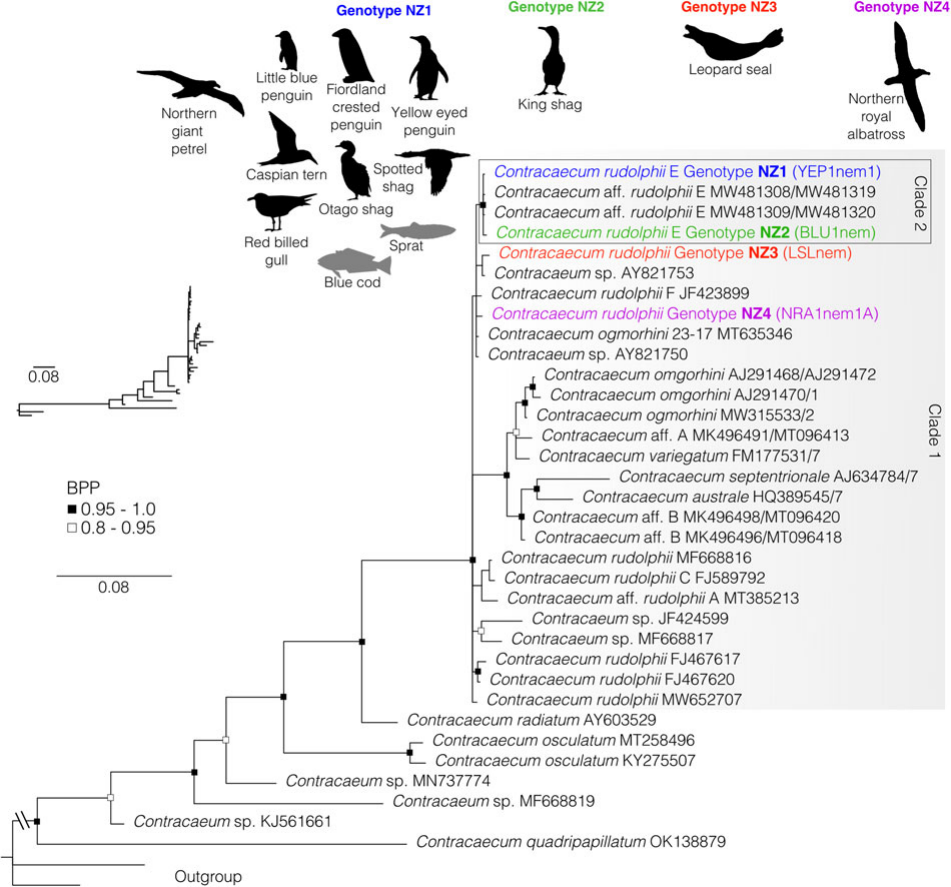
Bayesian phylogenetic inference of nematodes of the genus *Contracaecum* (family Anisakidae) inferred from ITS1 and ITS2 data. Black silhouettes represent definitive hosts and grey silhouettes represent intermediate hosts. BPP denoted by black and black-outlined white squares. Each colour represents a unique genotype recovered in this survey from New Zealand's marine animals. Scale represents substitution per base. Outgroups include representatives of genus *Hysterothylacium* (MW370746 and MF680035).

**Fig. 6.**
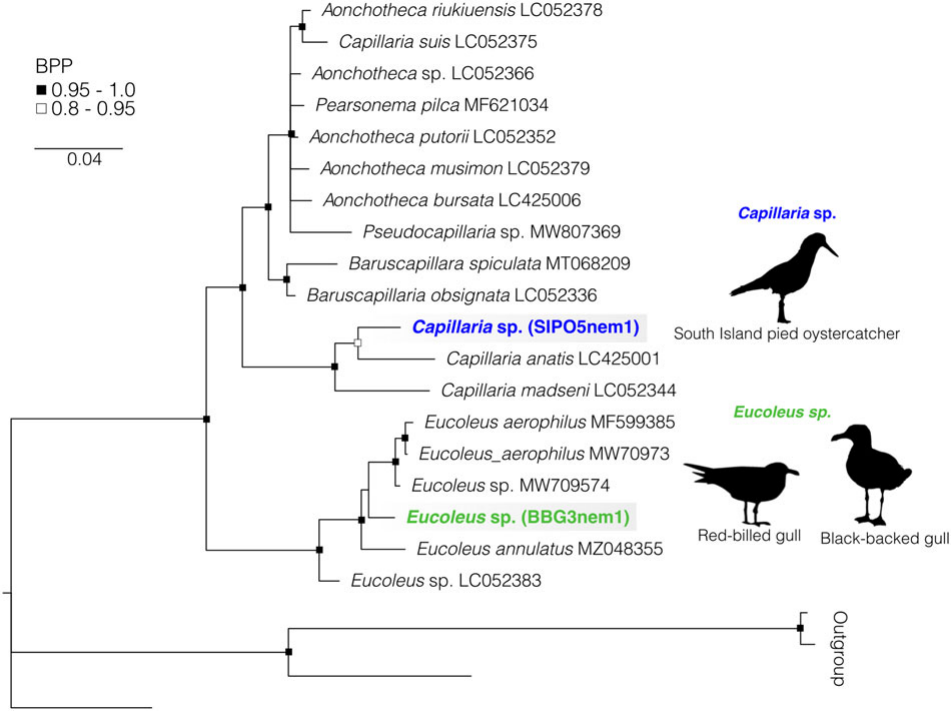
Bayesian phylogenetic inference of nematodes from family Capillariidae inferred from 18S data. Black silhouettes represent definitive hosts. Each colour represents a species recovered in this survey from New Zealand's marine animals. BPP denoted by black and black-outlined white squares. Scale represents substitution per base. Outgroups include representatives of Trichuridae, Trichinellidae and Habronematidae (AY851261, AY851265, AY702701 and EU004816).

In total, we found that 66% of host species investigated (62 out of 94) were infected with parasitic nematodes, and identified 23 species within 7 families (Acuariidae, Desmidocercidae, Hedruridae, Ascarididae, Anisakidae, Cucullanidae and Capillariidae) from 2 suborders (21 species of 7 families belonging to suborder Spirurina and 2 species of 1 family belonging to suborder Trichinellina) within phylum Nematoda. Nematodes were recovered from a range of infection sites, from lungs, gastrointestinal tracts, muscle tissues and other internal organs, including within 70% (28/40) of teleost fish species, 77% (30/39) of seabird species, 40% (4/10) of chondrichthyan species, 50% (2/4) cephalopod species and within the 1 marine mammal species investigated. The number of nematode species per infected host species ranged from 1 to 5 and on average each infected species hosted 1.5 nematodes. Of the fish host species investigated, mullet and sprat sp. 1 were infected with the highest number of nematode species, 4 and 5 species, respectively. The nematodes infecting seabirds ranged from 1 to 4 species per infected host (on average, 1.9 species per host), and red-billed gulls, spotted shags and white-chinned petrels all hosted 4 species each. All chondrichthyans, the leopard seal and all infected cephalopods hosted 1 nematode species each. Table 1 lists each of the nematode–host associations recovered in this study, including life stage data, host scientific names and which associations constitute new host records. Records identified using morphological data only are also included in this table. Below we present estimated phylogenies for each main clade of nematodes infecting New Zealand's marine animals, including suborder Spirurina (Fig. 1), family Acuariidae (Fig. 2), genera *Hysterothylacium* (Fig. 3), *Anisakis* (Fig. 4) and *Contracaecum* (Fig. 5) and family Capillariidae (Fig. 6) to illustrate the genetic diversity uncovered.

**Table 1.** List of parasitic nematode species recovered from New Zealand's marine animals in this study including data regarding new geographic records, life stage (A = adult, L = larval), host and if the host–nematode association is new for New Zealand's exclusive economic zone (NZ EEZ)

Nematode species	New geographic record for NZ EEZ?	Life stage	Host common name	Host species	Reference
*Diomedenema dinarctos* Presswell and Bennett, 2021*a*, 2021*b*	No	A	Fiordland crested penguin	*Eudyptes pachyrhynchus*	Presswell and Bennett (2021*a*)
		A	Snares crested penguin	*Eudyptes robustus*	New record
*Desmidocercella australis* Mawson, 1953	No	A	Spotted shag	*Phalacrocorax punctatus*	Presswell and Bennett (2021*b*)
*Hedruris spinigera* Baylis, 1931	No	A	Sprat sp. 1	*Sprattus antipodum*	New record
		A	Mullet	*Aldrichetta forsteri*	Luque *et al.* (2010)
*Neoterranova* sp.	Yes	A	Sixgill shark	*Hexanchus griseus*	New record
		A	Spiny dogfish	*Squalus acanthias*	New record
*Porrocaecum* sp.	Unknown	A	Black-backed gull	*Larus dominicanus*	New record
*Porrocaecum reticulatum* Linstow, 1899	Yes	A	Kingfisher	*Todiramphus sanctus*	New record
*Dichelyne pleuronectidis* (Yamaguti, 1935)	Yes	A	NZ sole	*Peltorhamphus novaezeelandiae*	New record
Cucullanidae gen. sp.	Unknown	A	Giant stargazer	*Kathetostoma giganteum*	New record
*Cosmocephalus jaenschi* Johnston and Mawson, 1941	No	A	Spotted shag	*P. punctatus*	Presswell and Bennett (2021*b*)
		A	Little pied shag	*Microcarbo melanoleucos*	Presswell and Bennett (2021*b*)
		A	Red-billed gull	*Chroicocephalus scopulinus*	New record
		A	Black-backed gull	*L. dominicanus*	New record
		L	Sprat sp. 1	*S. antipodum*	New record
		L	Triplefin spp.	Tripterygiidae gen. spp.	New record
*Stegophorus macronectes* (Johnston and Mawson, 1942)	No	A	Northern royal albatross	*Diomedea sanfordi*	New record
		A	Little blue penguin	*Eudyptula novaehollandiae*	Bennett *et al*. (2021)
		A	Common diving petrel	*Pelecanoides urinatrix*	New record
		A	Buller's mollymawk	*Thalassarche bulleri*	New record
		A	Salvin's mollymawk	*Thalassarche salvini*	New record
		A	White-chinned petrel	*Procellaria aequinoctialis*	New record
*Ingliseria cirrohamata* (Linstow, 1888)	No	A	Spotted shag	*P. punctatus*	Presswell and Bennett (2021*b*)
		A	Variable oystercatcher	*Haematopus unicolor*	New record
		A	Red-billed gull	*C. scopulinus*	New record
		A	Otago shag	*Leucocarbo chalconotus*	Presswell and Bennett (2021*b*)
		A	Little pied shag	*M. melanoleucos*	Presswell and Bennett (2021*b*)
*Seuratia shipleyi* (Stossich, 1900)	Yes	A	Salvin's mollymawk	*T. salvini*	New record
		A	White-chinned petrel	*P. aequinoctialis*	New record
		A	Flesh-footed shearwater	*Ardenna carneipes*	New record
		A	Grey-headed mollymawk	*Thalassarche chrysostoma*	New record
		A	White-headed petrel	*Pterodroma lessonii*	New record
		A	Sooty shearwater	*Ardenna grisea*	New record
		A	Black petrel	*Procellaria parkinsoni*	New record
		A	Westland petrel	*Procellaria westlandica*	New record
		A	Buller's mollymawk	*T. bulleri*	New record
		A	Grey petrel	*Procellaria cinerea*	New record
*Pectinospirura argentata* Wehr, 1933	Yes	A	Royal spoonbill	*Platalea flavipes*	New record
			Black-backed gull	*L. dominicanus*	New record
*Viktorocara torea* Clark, 1978	No	A	Variable oystercatcher	*H. unicolor*	New record
*Hysterothylacium aduncum* (Rudolphi, 1802)	No	A	Blue warehou	*Seriolella brama*	Brunsdon, unpublished (1956)
		A	Silver warehou	*Seriolella punctata*	Korotaeva (1975)
		A	Tarahiki	*Nemadactylus macropterus*	Vooren and Tracey (1976)
		A	School shark	*Galeorhinus galeus*	Brunsdon, unpublished (1956)
		A	Red gurnard	*Chelidonichthys cuculus*	Brunsdon, unpublished (1956)
		A	Red cod	*Pseudophycis bachus*	Brunsdon, unpublished (1956)
		A	Rough skate	*Zearaja nasuta*	Brunsdon, unpublished (1956)
		L	Lemon sole	*Pelotretis flavilatus*	New record
		L	Witch	*Arnoglossus* sp.	Brunsdon, unpublished (1956)
		L	Mullet	*A. forsteri*	Brunsdon, unpublished (1956)
		L	Crested bellowsfish	*Notopogon lilliei*	Brunsdon, unpublished (1956)
		L	Scaly gurnard	*Lepidotrigla brachyoptera*	Brunsdon, unpublished (1956)
		L	Clingfish	*Gastroscyphus hectoris*	New record
		L	Triplefin spp.	Tripterygiidae gen. spp.	New record
		L	Pigfish	*Congiopodus leucopaecilus*	Brunsdon, unpublished (1956)
		L	Sprat sp. 1	*S. antipodum*	New record
		L	Sprat sp. 2	*Sprattus muelleri*	New record
		L	Anchovy	*Engraulis australis*	New record
		L	Seahorse	*Hippocampus abdominalis*	New record
		L	Opalfish	*Hemerocoetes monopterygius*	Brunsdon, unpublished (1956)
*Hysterothylacium deardorffoverstreetorum* Knoff, Felizardo, Iñiguez, Maldonado, Torres, Magalhães Pinto and Gomes, 2012	Yes	A	Giant stargazer	*K. giganteum*	New record
		L	Triplefin sp. 2	*Forsterygion capito*	New record
*Anisakis* sp. 1 (haplotype NZ1)	Unknown	L	Warty squid	*Moroteuthopsis ingens*	Bennett *et al*. (2022*b*); *Anisakis nascettii* recovered from this host (Mattiucci *et al*., 2009; 2018)
*Anisakis simplex* s.l.	No	L	Salvin's mollymawk	*T. salvini*	New record
		L	White-chinned petrel	*P. aequinoctialis*	New record
		L	Northern giant petrel	*Macronectes halli*	New record
		L	White-capped mollymawk	*Thalassarche cauta*	New record
		L	Scarlett wrasse	*Notolabrus celidotus*	Brunsdon, unpublished (1956)
		L	Mullet	*A. forsteri*	Brunsdon, unpublished (1956)
		L	Arrow squid	*Nototodarus sloanii*	Smith *et al*. (1981); Wharton *et al*. (1999); Bennett *et al*. (2022*b*)
		L	Barracouta	*Thyrsites atun*	Wierzbicka and Gajda (1984); Hurst (1984) Brunsdon, unpublished (1956); Mehl (1970); *Anisakis pegreffii* recovered from this host (Mattiucci *et al*., 1997, 2018)
		L	Tarahiki	*N. macropterus*	Vooren and Tracey (1976)
		L	Silver warehou	*S. punctata*	Korotaeva 1975)
		L	Blue cod	*Parapercis colias*	Brunsdon, unpublished (1956); *Anisakis pegreffii* and *Anisakis berlandi* recovered from this host (Mattiucci *et al*., 1997, 2018)
		L	Pigfish	*C. leucopaecilus*	New record
		L	Olive rockfish	*Acanthoclinus fuscus*	New record
		L	Sprat sp. 2	*S. muelleri*	New record
		L	Banded wrasse	*Pseudolabrus fucicola*	New record
		L	Thornfish	*Bovichtus variegatus*	New record
*Anisakis* sp. indet.	Unknown	L	Flesh-footed shearwater	*A. carneipes*	New record
		L	Sooty shearwater	*A. grisea*	New record
		L	Variable oystercatcher	*H. unicolor*	New record
		L	Westland petrel	*P. westlandica*	New record
		L	King shag	*Leucocarbo carunculatus*	New record
		L	Black petrel	*P. parkinsoni*	New record
		L	Grey petrel	*P. cinerea*	New record
		L	Cape petrel	*Daption capense*	New record
		L	Common diving petrel	*P. urinatrix*	New record
		L	Triplefin spp.	Tripterygiidae gen. spp.	New record
		L	Common roughy	*Paratrachichthys trailli*	Brunsdon, unpublished (1956)
		L	Red gurnard	*C. cuculus*	Brunsdon, unpublished (1956)
		L	Blue warehou	*S. brama*	Brunsdon, unpublished (1956)
*Anisakis* sp. 2 (haplotype NZ23)	Unknown	L	White-chinned petrel	*P. aequinoctialis*	New record
*Contracaecum rudolphii* E Shamsi *et al*. 2009*a*	No	A	Fiordland crested penguin	*E. pachyrhynchus*	Presswell and Bennett (2021*a*)
		A	Little blue penguin	*E. novaehollandiae*	Bennett *et al*. (2021)
		A	Spotted shag	*P. punctatus*	Presswell and Bennett (2021*b*)
		A	Otago shag	*L. chalconotus*	Presswell and Bennett (2021*b*)
		A	Caspian tern	*Hydroprogne caspia*	New record
		A	Red-billed gull	*C. scopulinus*	New record
		A	Yellow-eyed penguin	*Megadyptes antipodes*	Ranum and Wharton (1996)
		A	King shag	*L. carunculatus*	New record
		A	Northern giant petrel	*M. halli*	New record
		L	Blue cod	*P. colias*	New record
		L	Sprat sp. 1	*S. antipodum*	New record
*Contracaecum rudolphii* Hartwich, 1964	No	A	Leopard seal	*Hydrurga leptonyx*	New record
		A	Northern royal albatross	*D. sanfordi*	New record
		A	King shag	*L. carunculatus*	New record
*Eucoleus* sp.	Yes	A	Black-backed gull	*L. dominicanus*	New record
		A	Red-billed gull	*C. scopulinus*	New record
*Capillaria* sp.	Unknown	A	South Island pied oystercatcher	*Haematopus finschi*	New record

Geographic records entered as ‘Unknown’ are those where we cannot be certain that our specimens belong to the same taxon as a similar one mentioned in the literature, or those that require further study to confirm their identity.

### Suborder Spirurina

Our estimated 18S phylogeny of suborder Spirurina included 17 unique newly generated sequences, 3 outgroups and 71 downloaded sequences representing 6 infraorders within Spirurina, with some clades collapsed for clarity (Fig. 1). The collapsed clades comprise 16 Acuariid (5 newly generated and 11 downloaded: accessions presented in Fig. 2), 4 *Hysterothylacium* (2 newly generated and 2 downloaded: MF072709 and MF072705), 3 *Anisakis* (1 newly generated and 2 downloaded: MT246663 and MF072697) and 3 *Contracaecum* (1 newly generated and 2 downloaded: MT233442 and AY702702) sequences. The Oxyuridomorpha, Gnathostomatomorpha and Rhigonematomorpha were found to be monophyletic, and the positions of Oxyuridomorpha and Gnathostomatomorpha are strongly supported in the estimated phylogeny, congruent with what has previously been found for these groups (e.g. Laetsch *et al*., 2012) (Fig. 1).

Infraorder Spiruromorpha is displayed as paraphyletic in this reconstruction, comprising 3 clades within Spirurina, 2 of which form polytomies with members of Spirurina incertae sedis. Within the first Spiruromorpha clade, we recovered Desmidocercidae and Acuariidae representatives (Acuariidae displayed in Fig. 2). Within Desmidocercidae, the *Diomedenema dinarctos* 18S sequences produced here are identical to the one produced in the original species description by Presswell and Bennett (2021*a*) from Fiordland crested penguins (GenBank MW718120). Here, we provide additional gene sequences for the species, including *cox*1 and ITS1 (Supplementary material 2). Our *Desmidocercella australis* sequence also matched one produced by Presswell and Bennett (2021*b*) from spotted shags (GenBank MW481211).

Within family Hedruridae (infraorder Spirurina), *Hedruris spinigera* was found infecting sprat and mullet, and the sequences produced matched 100% to that of *H. spinigera* infecting amphipods in New Zealand from Luque *et al*. (2010).

Infraorder Ascaridomorpha occupies a polyphyletic position in our estimated phylogeny forming 2 separate clades, only 1 of which (clade including superfamily Ascaridoidea) showed high nodal support for its positioning as a monophyletic group within Spirurina (Fig. 2). Within superfamily Ascaridoidea, we recovered species belonging to genus *Hysterothylacium* (family Raphidascarididae), depicted in Fig. 3. We also recovered species from family Anisakidae, genus *Neoterranova* infecting elasmobranchs and genus *Porrocaecum* infecting seabirds (Fig. 1). Species recovered that belong to genera *Anisakis* and *Contracaecum* are detailed below and depicted in Figs 4 and 5, respectively.

The second unresolved Ascaridomorpha clade includes representatives of families Cosmocercidae (superfamily Cosmocercoidea), Quimperidae and Cucullanidae (superfamily Seuratoidea), of which we recovered 2 cucullanid species infecting fish definitive hosts (Fig. 1). Intraspecific uncorrected pairwise genetic divergence between our *Dichelyne pleuronectidis* sequence and that of the same species isolated from ridged-eye flounder caught in the South China Sea (GenBank KJ855210; Li *et al*., 2014) is 2.47%. The genetic divergence threshold for delineation of species within *Dichelyne* is estimated at 2.04–3.39% meaning it is not possible to confirm whether or not these 2 sequences are indeed the same species (Li *et al*., 2014).

### Family Acuariidae

Acuariidae was the most diverse nematode family recovered from New Zealand's marine animals; all acuariids infect seabirds as definitive hosts, and all were found infecting at least 2 seabird species each. Overall, we recovered 6 species of acuariids from seabirds, all of which are depicted in Fig. 2, except for *Viktorocara torea* for which DNA amplification was not successful.

Our resulting 18S acuariid phylogeny includes 7 newly generated sequences and 13 sequences from the literature (including 2 outgroup sequences). It reflects the monophyletic nature of Acuariidae with high nodal support (as depicted in previous studies, e.g. Černotíková *et al*., 2011; Mutafchiev *et al*., 2020) (Fig. 2). Our 18S tree provides high support for the positioning of *Pectinospirura argentata* as a sister taxon to most other acuariid representatives, as well as the positioning of *Seuratia shipleyi* as a distinct sister taxon to most other acuariids (Fig. 2). Only 1 species recovered (*S. shipleyi*) appeared to exhibit intraspecific genetic variation at both 18S (genetic divergence 0.1%) and 28S (genetic divergence 0.8%; see Supplementary material 2 for GenBank accession numbers).

### Genus *Hysterothylacium*

*Hysterothylacium* spp. were found infecting teleost fish as intermediate hosts, and teleosts and chondrichthyans as definitive hosts. We recovered 2 species, *Hysterothylacium aduncum* and *Hysterothylacium deardorffoverstreetorum*, of which *H. deardorffoverstreetorum* was only recovered from 1 definitive (giant stargazer) and 1 intermediate host (triplefin), whereas *H. aduncum* was found in 19 teleost and elasmobranch host species. Our ITS1 and ITS2 phylogeny exhibited high nodal support and resolution for each branch split (Fig. 3). Our sequences of *H. aduncum* were all identical showing no intraspecific variation in ITS1 and ITS2. *Hysterothylacium aduncum* sequences produced here exhibited 2.1% genetic divergence in ITS 1 and ITS2 from a specimen of *H. aduncum* from a whitespotted conger eel off the coast of China (GenBank MF539777). Although only 2 sequences were produced for *H. deardorffoverstreetorum* (1 from each host), they also showed no intraspecific genetic variation at ITS1 and ITS2. This species exhibited 1.15% genetic divergence from the closest representative on GenBank; *H. deardorffoverstreetorum* sequenced from a flounder off the coast of Brazil (GenBank MF539777; Knoff *et al*., 2012). The mean genetic divergence between species within *Hysterothylacium* is 28.7% for ITS1 and ITS2.

### Genus *Anisakis*

*Anisakis* spp. were recovered from New Zealand fish and cephalopod intermediate hosts, and seabird accidental hosts. Based on our estimated phylogeny of *cox*1 data, we recovered 3 genetic clades comprising 24 unique haplotypes (Fig. 4). At the *cox1* level, our phylogeny showed little support and low resolution for the interrelationships among species within *Anisakis* (Fig. 4). However, high support is provided for some within-species groupings. *Anisakis* sp. 1 (haplotype NZ1) was recovered from warty squid in the Chatham Rise, off the east coast of New Zealand. Warty squid were examined off the coast of Australia by Mattiucci *et al.* (2009), who genetically characterized the infections as *Anisakis nascettii*, although no *A. nascettii* are currently available for *cox1*. The sequences with the highest similarity to *Anisakis* sp. 1 from warty squid belong to haplotypes NZ2–12 and *Anisakis pegreffii* from a chub mackerel from the coast of Japan (GenBank LC222461; Yamada *et al*., 2017). These sequences showed 5.47 and 6.16% divergence respectively with *Anisakis* sp. 1. The second recovered species complex, *Anisakis simplex* sensu lato (s.l.) (haplotypes NZ2–22) was recovered from the viscera of a range of fish intermediate hosts (Fig. 5), and within the internal cavity of arrow squid as L3 encysted larvae. A range of seabirds were accidental hosts to *A. simplex* s.l., in which immature *Anisakis* were found in the gastrointestinal tract, typically the oesophagus or stomach. The intraspecific genetic divergence between *cox*1 haplotypes NZ2–22 all belonging to *A. simplex* s.l. was 0.98%, and the divergence between all newly sequenced *A. simplex* s.l. and closest relatives in the clade was 0.96%. The closest relatives on GenBank included in this clade are *A. pegreffii* sequence from a chub mackerel off the coast of Japan (GenBank LC222461), *Anisakis physeteris* from the Taiwan Strait (GenBank GU112207), *A. simplex* from a conger eel in South Korea (GenBank AY994157) and *Anisakis typica* from cetaceans in the Philippines (GenBank KJ786265 and KF356669). The third species recovered in this survey is *Anisakis* sp. 2 (haplotype NZ23) which was recovered from a white-chinned petrel accidental host. Our *Anisakis* sp. 2 haplotype NZ23 is well supported as a sister taxon to sequences of *A. physeteris*, with a mean genetic divergence of 4.04% between the 2 groups. The closest *A. physeteris* to our recovered *Anisakis* sp. 2 were from mahi-mahi and skipjack tuna off the southern coast of California, USA (GenBank MF663247-9 and MF752458). The mean genetic divergence between *Anisakis* sp. 1 and *A. simplex* s.l. sequences was 6.27%, between *A. simplex* s.l. and *Anisakis* sp. 2 it was 15.90% and between *Anisakis* sp. 1 and *Anisakis* sp. 2 it was 15.52% at the *cox*1 level. A few specimens that were successfully sequenced for 18S, but not *cox1*, could not be placed in the phylogeny nor identified further than genus level. These specimens, *Anisakis* sp. indet., were found infecting multiple accidental and intermediate hosts (Fig. 4).

### Genus *Contracaecum*

In our ITS1 and ITS2 phylogeny of species within genus *Contracaecum*, our newly generated sequences grouped together with other sequences of *Contracaecum* in an unresolved polytomy (Fig. 5). We recovered 4 unique genotypes, of which 1 (genotype NZ1) was the most commonly recovered from marine animals in New Zealand. This was identified as *Contracaecum rudolphii* E (as defined by Shamsi *et al*. (2009*b*) from Australian seabirds) and we recovered specimens from 7 seabird species. L3 larvae of this genotype were also recovered from 2 teleost intermediate hosts. These sequences were identical to those presented in Presswell and Bennett (2021*b*) from spotted and little pied shags in Otago, New Zealand (MW481308/MW481319 and MW481309/MW481320). Also positioned in this clade is the second genotype of *C. rudolphii* E (NZ2) which was recovered from king shag regurgitates from the Marlborough Sounds, New Zealand. This genotype exhibited only 0.13% genetic divergence from genotype NZ1 and other *C. rudolphii* E sequences. The third genotype of *C. rudolphii* (genotype NZ3) was recovered from the gastrointestinal tract of a leopard seal which was found on the Otago coast. The closest sequences are 2 *Contracaecum* sp. sequences from a California sea lion in California, USA (GenBank AY821753 and AY821750) which were 0.24 and 0.25% divergent, respectively. The last genotype of *C. rudolphii* was recovered from the gastrointestinal tract of a northern royal albatross caught as fisheries trawl by-catch somewhere in New Zealand's EEZ (genotype NZ4). This genotype was also closest to *Contracaecum* sp. from a California sea lion in the USA (GenBank AY821750) and *Contracaecum ogmorhini* from Tiger flathead on the southeast coast of Australia (GenBank MT635346; Hossen *et al*., 2021) which were both 0.22% divergent at the ITS marker level. The genetic divergence between the *C. rudolphii* E clade and leopard seal *C. rudolphii* is 1.25%. The average genetic divergence between each species clade was 0.2–17.8%.

### Family Capillariidae

Family Capillariidae (order Trichinellida: suborder Trichinellina) is the only family infecting New Zealand's marine animals found in this study that does not belong to suborder Spirurina. We recovered 2 species infecting 3 seabird definitive host species. The interrelationships and nodal support among species and genera within family Capillariidae follow closely those of Borba *et al*. (2019) (Fig. 6). Our phylogeny supports the monophyly of the genera *Eucoleus* and *Capillaria* as reported in previous studies using the 18S gene (Tamaru *et al*., 2015; Borba *et al*., 2019; Garbin *et al*., 2021). The exception is *Capillaria suis* which appeared nested within *Aonchotheca* species, as in Borba *et al*. (2019).

### New host and distribution records

We provide data for 70 new nematode–host associations, and provide new geographic records for 7 of the 23 nematode species recovered that were previously unknown in New Zealand EEZ (Table 1).

We update the taxonomy of previously unresolved parasite species involved in 16 nematode–host associations. Records of larval *Hysterothylacium* sp. in the literature can be confirmed here using genetic data as belonging to *H. aduncum*. This includes for hosts red cod, red gurnard, school sharks, witch, mullet, opalfish and crested bellowsfish. Existing records of *Anisakis* sp. can also be updated as belonging to *A. simplex* s.l., including for scarlet wrasse, mullet, blue cod, tarakihi and silver warehou. We provide corrected taxonomic records for some species previously only reported to the genus level. Some literature references to *Contracaecum* sp. are updated to *C. rudolphii* E (genotype NZ1). This includes data for yellow-eyed penguins, little blue penguins (correction of the name *Contracaecum eudyptulae* of Bennett *et al*., 2021, possibly synonymous with *C. rudolphii* E) and spotted and Otago shags (in Presswell and Bennett, 2021*b*).

We provide the first records of a parasitic nematode infection for 24 of the total 94 host species dissected (25% of host species investigated). For teleosts, we present the first reported nematodes infecting the following fishes: sprat sp. 1, sprat sp. 2, triplefin spp., clingfish, seahorse, anchovy, pigfish, olive rockfish, banded wrasse and thorn fish. For seabirds, we present the first record of parasitic nematodes infecting Snares crested penguin, kingfisher, Salvin's mollymawk, flesh-footed shearwater, common diving, Westland, Cape, Northern giant, white-chinned and white-headed petrels, spoonbill, variable oystercatcher, Caspian tern and king shag.

## Discussion

This study represents the first large-scale, genetically based biodiversity survey of parasitic nematodes from a wide range of host taxa in New Zealand's marine environment, and possibly the first anywhere in the world for a whole marine ecosystem. Our results shed light on patterns of host use across all parasitic nematodes within a single ecosystem. We uncovered a high phylogenetic diversity of nematodes living within New Zealand's marine animals, comprising 23 species belonging to 7 families. Most vertebrate species dissected hosted at least 1 species of nematode (those that did not were typically represented by low sample sizes). We report 70 new host and 7 new geographic records and update the taxonomic records for 16 host–nematode associations. These results provide baseline data of host–nematode interactions, nematode genetic diversity and host specificity in New Zealand's marine animals. Some species recovered here are potentially zoonotic, pathogenic or otherwise worth noting. We hope that these data will serve as a starting point for future comparative studies pertaining to how nematode biodiversity and distributions change in response to natural and anthropogenic pressures.

The most commonly recovered nematodes in this survey were those belonging to *Anisakis*, which infected 35% of fish and 24% of seabird species. This high occurrence is not surprising considering that species within family Anisakidae are the most commonly reported nematodes infecting marine animals globally, primarily due to their ubiquitous distribution and sometimes zoonotic pathogenicity. The interrelationships between and within species of *Anisakis* have been somewhat clarified in recent years with the application of molecular techniques, which have uncovered a number of cryptic species and species complexes (e.g. Mattiucci *et al*., 1997, 2005, 2014, 2018; Abollo *et al*., 2003; Shamsi *et al*., 2012). The difficulty of species discrimination by morphology, especially when larval stages are recovered, makes genetic data essential for understanding which genotypes or species cause zoonotic infections (Mattiucci *et al*., 2011) and which may have pathogenic effects on their non-human hosts (Shamsi *et al*., 2012). In this study, within New Zealand, we recovered over 20 new unique *cox1* haplotypes not previously sequenced which can be accessed by future researchers to better understand this group of nematodes.

Fewer than a quarter of New Zealand seabird species have records of parasitic infections (Bennett *et al*., 2022*a*). Furthermore, a higher proportion of non-threatened seabirds have parasite records compared to threatened species (Bennett *et al*., 2022*a*). This constitutes a large knowledge gap for seabird conservation as evidence from around the world suggests nematodes are often associated with, or contribute to, decreased host health and survival (e.g. Abollo *et al*., 2001*b*; Schramm *et al*., 2018; Vanstreels *et al*., 2018). Many seabirds dissected in this study are native, endemic, threatened or hold cultural significance in New Zealand, such as the yellow-eyed penguin and northern royal albatross. These animals are difficult to obtain for research purposes; there are few of them and they are often used for other purposes post-mortem (e.g. cultural burials). Therefore, the specimens opportunistically sampled here have greatly increased our knowledge regarding nematode infections in New Zealand's seabirds. For example, our dataset includes the first record of any parasitic helminth infecting a New Zealand petrel or shearwater species, of which there are currently 27 and 7 species, respectively. Considering that threatened seabird species are more likely to experience extinction events than non-threatened species, pre-emptive nematode data as reported here may provide a first point of call for the conservation of seabirds when or if disease strikes.

We recovered some potentially disease-causing nematodes. *Diomedenema dinarctos* described by Presswell and Bennett (2021*a*) infecting Fiordland crested penguin was recovered in this survey from both Fiordland crested and Snares crested penguins, constituting a new host record for the latter. This nematode can cause severe haemorrhaging, air sacculitis and sometimes death (e.g. Vanstreels *et al*., 2018), therefore this discovery represents a significant find for the conservation of Snares crested penguins, and warrants investigation for this nematode in other penguins, such as yellow-eyed penguin, the world's most threatened penguin species (Robertson *et al*., 2017).

Of the 45 genera currently recognized in family Acuariidae (Bain *et al*., 2014), 6 are reported in New Zealand's seabirds. Of those, 4 genera represent new geographic records for New Zealand. For example, *P. argentata* was previously known from South and North America, but never in the Pacific. We extend its known distribution to New Zealand, where it infects black-backed gulls and spoonbills. It is not possible to make inferences regarding whether our newly recovered geographic records signify a change in distribution over time, or simply if these New Zealand seabird species were already hosts and *P. argentata* had thus far not been reported. Such questions could be posed for each of the 7 nematode species newly reported for New Zealand herein. These data can, however, provide a starting point to test hypotheses regarding temporal and spatial patterns of nematode infections in future.

The findings of this study, although resulting from a large sampling effort, nonetheless likely do not reflect the total biodiversity of parasitic nematodes in the study area. New Zealand is thought to host at least 106 zooparasitic nematode taxa according to data gathered for a forthcoming review of its marine biota (B. Presswell, personal communication), while only 23 taxa were recovered here. Most records in the literature rely entirely on morphological data and revisiting such records with modern taxonomic techniques would be highly beneficial. In addition, many potential host species are yet to be investigated (Bennett *et al*., 2022*a*). Future research will be needed for more accurate estimates of overall parasitic nematode biodiversity.

New Zealand currently hosts at least 8 *Contracaecum* species from a range of seabirds and marine mammals. Of *Contracaecum* obtained from specimens inside New Zealand, only 1 of the 8 have been genetically characterized, although much research has been done in Australia for the Austral region (e.g. Shamsi *et al*., 2009*a*, 2009*b*, 2018). Here, we recovered only 1 species complex, i.e. *C. rudolphii*, consisting of 2 clades and 4 unique ITS genotypes. Although our results are likely somewhat spatially biased towards Otago in the South Island of New Zealand, many seabirds were collected from a nationwide fishery by-catch programme; if there were other *Contracaecum* present in New Zealand, it is likely we would have detected more species here. For example, in the literature it is expected that red-billed gulls host *Contracaecum microcephalum*, little blue penguins host *C. eudyptulae* and leopard seals would host *C. ogmorhini* although we recovered only *C. rudolphii* from each of those, albeit unique genotypes in each. Furthermore, our results suggest further investigation of *Contracaecum* in New Zealand is required for resolution of the group's diversity. Shamsi *et al*. (2009*a*, 2009*b*) inventoried *Contracaecum* species in some Australian birds using both molecular and morphological data, and a similar integrative taxonomic investigation into the genotypes recovered here would prove beneficial, especially considering the lack of genetic support in the ITS1 and ITS2 markers for the genotypes NZ3 and NZ4 recovered here (Fig. 5).

The phylogenetic relationships of nematode clades presented here represent only a small number of molecular markers, likely not 100% reflective of their true evolutionary history. Many groups remain relatively unresolved with low nodal support and discerning their phylogenetic relationships (especially within Spirurina) requires significantly greater species representation as well as additional genetic markers (Černotíková *et al*., 2011). Due to the large phylogenetic diversity of nematodes, inevitably a variety of molecular markers have been employed that are specific to each clade. As a result, some taxa are simply not comparable. For example, we used *cox*1 for species within *Anisakis* to be comparable with data from other researchers (e.g. Cross *et al*., 2007), although there are species within New Zealand sequenced using mtDNA *cox*2 data (Bello *et al*., 2021). Obtaining such additional gene sequences would also potentially allow for species level resolution.

Nematodes constitute a significant proportion of the world's biodiversity and deserve a proportionate effort to document their occurrence; if not for their contribution to biodiversity, then they should at least be inventoried for their impacts on host individuals and populations. This study uncovered a diverse range of nematode parasites living within some of New Zealand's marine animals and provided a molecular-based dataset which researchers can use for comparison in the future. Without baseline inventories such as this, our ability to understand how nematodes and their hosts will respond to natural and anthropogenic pressures would be greatly limited.

## Data Availability

All sequences generated in this study are available in GenBank under accessions: OP458396–OP458422, OP431140–OP431163, OP453417–OP453442, OP470829–OP470859, OP455795–OP455799, OP455085–OP455105, OP467567–OP467582 and OP503276–OP503310.

## Supplementary material

For supplementary material accompanying this paper visit https://doi.org/10.1017/S003118202200138X. click here to view supplementary material
