# Supplementary material for: Large-scale genetic investigation of nematode diversity and their phylogenetic patterns in New Zealand's marine animals
Source: Parasitology. 2022 Oct 6;149(13):1794–809. doi: 10.1017/S003118202200138X (PMC10090774; doi:10.1017/S003118202200138X)
Supplement: Supplementary file 1 [file S003118202200138Xsup.zip › S003118202200138Xsup001.docx]

Supplementary Material 1. Host species investigated for their nematode parasites in this study, their common names, number dissected and location of origin (where information is available). Fish taxonomy follows FishBase (Froese & Pauly 2022); bird taxonomy follows Avibase (Lepage 2019) and Rawlence et al. 2016; cephalopod taxonomy follows MolluscaBase.

| **Host group** | **Order/common name** | **Scientific name** | **N** | **Location** |
| --- | --- | --- | --- | --- |
| **Seabirds** |  |  |  |  |
|  | Anseriformes |  |  |  |
|  | NZ scaup | *Aythya novaeseelandiae* | 1 | Otago coast |
|  | Paradise shelduck | *Tardorna variegata* | 1 | Otago coast |
|  | Charadriiformes |  |  |  |
|  | Red-billed gull | *Chroicocephalus scopulinus* | 58 | Otago coast |
|  | South Island pied oystercatcher | *Haematopus finschi* | 4 | Otago coast |
|  | Variable oystercatcher | *Haematopus unicolor* | 6 | Otago coast |
|  | Pied stilt | *Himantopus himantopus* | 2 | Otago coast |
|  | Caspian tern | *Hydroprogne caspia* | 2 | Otago coast |
|  | Black-backed gull | *Larus dominicanus* | 46 | Otago coast |
|  | Ciconiiformes |  |  |  |
|  | Royal spoonbill | *Platalea flavipes* | 2 | Otago coast |
|  | Coraciiformes |  |  |  |
|  | Kingfisher | *Todiramphus sanctus* | 4 | Otago coast |
|  | Pelecaniformes |  |  |  |
|  | Otago shag | *Leucocarbo chalconotus* | 8 | Otago coast |
|  | King shag | *Leucocarbo carunculatus* | 1 | NZ EEZ |
|  | Foveaux shag | *Leucocarbo stewarti* | 1 | NZ EEZ |
|  | Little pied shag | *Microcarbo melanoleucos* | 7 | Otago coast |
|  | Spotted shag | *Phalacrocorax punctatus* | 34 | Otago coast |
|  | Procellariiformes |  |  |  |
|  | Flesh-footed shearwater | *Ardenna carneipes* | 5 | NZ EEZ |
|  | Sooty shearwater | *Ardenna grisea* | 17 | NZ EEZ |
|  | Cape petrel | *Daption capense* | 1 | NZ EEZ |
|  | Northern royal albatross | *Diomedea sanfordi* | 1 | NZ EEZ |
|  | Northern giant petrel | *Macronectes halli* | 2 | Otago coast |
|  | Fairy prion | *Pachyptila turtur* | 2 | Otago coast |
|  | Broad billed prion | *Pachyptila vittata* | 6 | Otago coast |
|  | Common diving petrel | *Pelecanoides urinatrix* | 1 | NZ EEZ |
|  | White-chinned petrel | *Procellaria aequinoctialis* | 11 | NZ EEZ |
|  | Grey petrel | *Procellaria cinerea* | 4 | NZ EEZ |
|  | Black petrel | *Procellaria parkinsoni* | 2 | NZ EEZ |
|  | Westland petrel | *Procellaria westlandica* | 25 | NZ EEZ |
|  | Cooks petrel | *Pterodroma cookii* | 1 | NZ EEZ |
|  | Mottled petrel | *Pterodroma inexpectata* | 2 | NZ EEZ |
|  | White-headed petrel | *Pterodroma lessonii* | 1 | NZ EEZ |
|  | Buller’s mollymawk | *Thalassarche bulleri* | 1 | NZ EEZ |
|  | White-capped mollymawk | *Thalassarche cauta* | 11 | NZ EEZ |
|  | Grey-headed mollymawk | *Thalassarche chrysostoma* | 1 | NZ EEZ |
|  | Salvin's mollymawk | *Thalassarche salvini* | 9 | NZ EEZ |
|  | Sphenisciformes |  |  |  |
|  | Fiordland crested penguin | *Eudyptes pachyrhynchus* | 6 | Otago coast |
|  | Snares crested penguin | *Eudyptes robustus* | 7 | Otago coast |
|  | Erect crested penguin | *Eudyptes sclateri* | 2 | Otago coast |
|  | Little blue penguin | *Eudyptula novaehollandiae* | 49 | Otago coast |
|  | Yellow-eyed penguin | *Megadyptes antipodes* | 6 | Otago coast |
| **Mammals** |  |  |  |  |
|  | Carnivora |  |  |  |
|  | Leopard seal | *Hydrurga leptonyx* | 2 | Otago coast |
| **Teleost fish** |  |  |  |  |
|  | Eupercaria *incertae sedis* |  |  |  |
|  | Spotted wrasse | *Notolabrus celidotus* | 1 | Otago coast |
|  | Banded wrasse | *Pseduolabrus fucicola* | 3 | Otago coast |
|  | Scarlett wrasse | *Pseudolabrus miles* | 7 | Otago coast |
|  | Ovalentaria *incertae sedis* |  |  |  |
|  | Olive rockfish | *Acanthoclinus fuscus* | 4 | Otago coast |
|  | Acropomatiformes |  |  |  |
|  | Slender stargazer | *Crapatalus angusticeps* | 1 | Otago coast |
|  | Beloniformes |  |  |  |
|  | Garfish | *Hyporhamphus ihi* | 2 | Otago coast |
|  | Blenniiformes |  |  |  |
|  | Triplefin sp. 2 | *Forsterygion capito* | 5 | Otago coast |
|  | Triplefin sp. 1 | *Forsterygion lapillum* | 7 | Otago coast |
|  | Triplefin sp. 3 | *Forsterygion* sp. | 1 | Otago coast |
|  | Triplefin spp. | Tripterygiidae gen. spp. | 23 | Otago coast |
|  | Centrarchiformes |  |  |  |
|  | Blue moki | *Latridopsis ciliaris* | 1 | Otago coast |
|  | Tarakihi | *Nemadactylus macropterus* | 13 | Otago coast |
|  | Clupeiformes |  |  |  |
|  | Anchovy | *Engraulis australis* | 5 | Otago coast |
|  | Sprat sp. 1 | *Sprattus antipodum* | 9 | Otago coast |
|  | Sprat sp. 2 | *Sprattus muelleri* | 15 | Otago coast |
|  | Gadiformes |  |  |  |
|  | Red cod | *Pseudophycis bachus* | 9 | Otago coast |
|  | Gobiesociformes |  |  |  |
|  | Clingfish | *Gastroscyphus hectoris* | 2 | Otago coast |
|  | Mugiliformes |  |  |  |
|  | Mullet | *Aldrichetta forsteri* | 9 | Otago coast |
|  | Ophidiiformes |  |  |  |
|  | Ling | *Genypterus blacodes* | 3 | Otago coast |
|  | Perciformes |  |  |  |
|  | Thornfish | *Bovichtus variegatus* | 1 | Otago coast |
|  | Red gurnard | *Chelidonichthys cuculus* | 7 | Otago coast |
|  | Pigfish | *Congiopodus leucopaecilus* | 9 | Otago coast |
|  | Stargazer | *Genyagnus monopterygius* | 3 | Otago coast |
|  | Opalfish | *Hemerocoetes monopterygius* | 2 | Otago coast |
|  | Giant stargazer | *Kathetostoma giganteum* | 4 | Otago coast |
|  | Scaly gurnard | *Lepidotrigla brachyoptera* | 6 | Otago coast |
|  | Blue cod | *Parapercis colias* | 5 | Otago coast |
|  | Pleuronectiformes |  |  |  |
|  | Witch | *Arnoglossus* sp. | 4 | Otago coast |
|  | Brill | *Colistium guentheri* | 3 | Otago coast |
|  | Lemon sole | *Pelotretis flavilatus* | 4 | Otago coast |
|  | NZ sole | *Peltorhamphus novaezeelandiae* | 4 | Otago coast |
|  | Sand flounder | *Rhombosolea plebeia* | 1 | Otago coast |
|  | Scombriformes |  |  |  |
|  | Kahawai | *Arripis trutta* | 1 | Otago coast |
|  | Blue warehou | *Seriolella brama* | 7 | Otago coast |
|  | Silver warehou | *Seriolella punctata* | 5 | Otago coast |
|  | Barracouta | *Thyrsites atun* | 10 | Otago coast |
|  | Syngnathiformes |  |  |  |
|  | Seahorse | *Hippocampus abdominalis* | 1 | Otago coast |
|  | Crested bellowsfish | *Notopogon lilliei* | 1 | Otago coast |
|  | Tetraodontiformes |  |  |  |
|  | Pufferfish | *Contusus richei* | 2 | Otago coast |
|  | Trachichthyiformes |  |  |  |
|  | Common roughy | *Paratrachichthys trailli* | 1 | Otago coast |
| **Chondrichthyans** |  |  |  |  |
|  | Carcharhiniformes |  |  |  |
|  | Carpet shark | *Cephaloscyllium isabella* | 3 | Otago coast |
|  | School shark | *Galeorhinus galeus* | 9 | Otago coast |
|  | Rig shark | *Mustelus lenticulatus* | 10 | Otago coast |
|  | Chimaeriformes |  |  |  |
|  | Elephantfish | *Callorhinchus milii* | 5 | Otago coast |
|  | Hexanchiformes |  |  |  |
|  | Sixgill shark | *Hexanchus griseus* | 2 | Chatham Rise |
|  | Sevengill shark | *Notorynchus cepedianus* | 1 | Otago coast |
|  | Rajiformes |  |  |  |
|  | Smooth skate | *Dipturus innominatus* | 2 | Chatham Rise |
|  | Rough skate | *Zearaja nasuta* | 4 | Otago coast |
|  | Squaliformes |  |  |  |
|  | Shortspine lanternshark | *Etmopterus unicolor* | 1 | Chatham Rise |
|  | Spiny dogfish | *Squalus acanthias* | 5 | Otago coast |
| **Cephalopods** |  |  |  |  |
|  | Octopoda |  |  |  |
|  | NZ octopus | *Macroctopus maorum* | 3 | Otago coast |
|  | Oegopsida |  |  |  |
|  | Arrow squid | *Nototodarus sloanii* | 10 | Otago coast |
|  | Warty squid | *Moroteuthopsis ingens* | 1 | Chatham Rise |
|  | Sepiida |  |  |  |
|  | Bob-tailed squid | *Sepioloidea pacifica* | 2 | Otago coast |
