# Supplementary material for: Large-scale genetic investigation of nematode diversity and their phylogenetic patterns in New Zealand's marine animals
Source: Parasitology. 2022 Oct 6;149(13):1794–809. doi: 10.1017/S003118202200138X (PMC10090774; doi:10.1017/S003118202200138X)
Supplement: Supplementary file 1 [file S003118202200138Xsup.zip › S003118202200138Xsup002.docx]

Supplementary Material 2. All newly produced gene sequences submitted to GenBank of nematodes infecting New Zealand marine animals resulting from this survey.

| **Taxon ID** | **Host species and isolate** |  | **GenBank Accession** | | | |
| --- | --- | --- | --- | --- | --- | --- |
|  |  | 18S | 28S | ITS1 | ITS2 | *cox*1 |
| *Diomedenema dinarctos* | Snares crested penguin  (SCPnem1) | OP458396 |  | OP470829 |  | OP431140 |
| *Desmidocercella australis* | Spotted shag  (SSHnem1) | OP458397 |  |  |  |  |
| *Hedruris spinigera* | Sprat sp. 1 (Fish1nem2_35) | OP458398 |  |  |  |  |
|  | Mullet (Mull4nem1_39) | OP458399 |  |  |  |  |
| *Neoterranova* sp. | Sixgill shark (Hex1nem1) | OP458400 |  |  |  |  |
|  | School shark (SPD1nem1_13) | OP458401 |  |  |  |  |
| *Porrocaecum* sp. | Black-backed gull (BBG23nem1) | OP458402 |  |  |  |  |
| *Porrocaecum reticulatum* | Kingfisher (KIN2nem1) | OP458403 |  |  |  |  |
| *Dichelyne pleuronectidis* | NZ sole (FF1nem1_10) | OP458404 |  |  |  |  |
| Cucullanidae gen. sp. | Giant stargazer (GIZ2nem2_41) | OP458405 |  |  |  |  |
| *Cosmocephalus jaenschi* | Red-billed gull (RGB45nem1) | OP458406 |  |  |  |  |
|  | Black-backed gull (BBGnem1) | OP458407 |  |  |  |  |
|  | Little pied shag (LSHnem1) | OP458408 | OP455795 |  |  |  |
|  | Spotted shag (SSHnem1) | OP458409 |  |  |  |  |
|  | Triplefin (TF2nem1_37) | OP458410 |  |  |  |  |
|  | Sprat sp. 1 (SPR1nem1_28) | OP458411 | OP455796 |  |  |  |
| *Stegophorus macronectes* | Northern royal albatross (NRA1nem2) | OP458412 |  |  |  |  |
|  | Little blue penguin (LBPnem1) | OP458413 |  |  |  |  |
|  | Common diving petrel (SB9nem2) | OP458414 |  |  |  |  |
| *Ingliseria cirrohamata* | Spotted shag (SSH12nem1) | OP458415 | OP455797 |  |  |  |
|  | Variable oystercatcher (VOC2nem1) | OP458416 |  |  |  |  |
| *Seuratia shipleyi* | White-chinned petrel (WLP5nem1) | OP458417 | OP455798 |  |  |  |
|  | Flesh-footed shearwater (FFSWAnem1) | OP458418 |  |  |  |  |
|  | Grey-headed mollymawk (GHM1nem1_16) | OP458419 |  |  |  |  |
|  | Salvin’s mollymawk (SA5nem1) | OP458420 | OP455799 |  |  |  |
| *Pectinospirura argentata* | Black-backed gull (BBG31nem1) | OP458421 |  |  |  |  |
|  | Spoonbill (SPB2nem1) | OP458422 |  |  |  |  |
| *Hysterothylacium aduncum* | Blue warehou (WAR1nem1_41) | OP455085 |  | OP470830 | OP453417 |  |
|  | Red cod (RCDEnem3_41) | OP455086 |  | OP470831 | OP453418 |  |
|  | Silver warehou (SWH2nem1) | OP455087 |  | OP470832 | OP453419 |  |
|  | Red gurnard (GUR2nem2_41) | OP455088 |  | OP470833 | OP453420 |  |
|  | School shark (SCH3nem1) | OP455089 |  |  | OP453421 |  |
|  | Tarakihi (TERA7nem1_41) | OP455090 |  | OP470834 | OP453422 |  |
|  | Opalfish (OPF1nem1_17) | OP455091 |  |  |  |  |
|  | Seahorse (SH1nem1) | OP455092 |  | OP470835 |  |  |
|  | Clingfish (CLI1nem1_18) | OP455093 |  | OP470836 | OP453423 |  |
|  | Lemon sole (FF2anem1_10) | OP455094 |  | OP470837 |  |  |
|  | Witch (WIT1nem1) | OP455095 |  | OP470838 | OP453424 |  |
|  | Mullet (Mull9nem1_41) | OP455096 |  | OP470839 | OP453425 |  |
|  | Crested bellowsfish (CBFnem1) | OP455097 |  | OP470840 | OP453426 |  |
|  | Scaly gurnard (SCG4nem1_41) | OP455098 |  | OP470841 | OP453427 |  |
|  | Triplefin spp. (Fish2nem1) | OP455099 |  | OP470842 |  |  |
|  | Pigfish (PIG1nem1_10) | OP455100 |  |  | OP453428 |  |
|  | Anchovy (ANC4nem1_41) | OP455101 |  | OP470843 |  |  |
|  | Sprat sp. 1 (SPR5nem1_28) | OP455102 |  | OP470844 | OP453429 |  |
|  | Sprat sp. 2 (SPM1nem1_41) | OP455103 |  | OP470845 | OP453430 |  |
| *Hysterothylacium deardorffoverstreetorum* | Giant stargazer (GIZnem1_41) | OP455104 |  | OP470846 |  |  |
|  | Triplefin sp. 2 (TF3nem1_21) | OP455105 |  | OP470847 | OP453431 |  |
| *Anisakis* sp. 1 (NZ1) | Warty squid (MIQ1nem1_10) | OP503276 |  |  |  | OP431141 |
| *Anisakis simplex s.l.* (NZ2) | Barracouta (BAR1nem1_17) | OP503277 |  |  |  | OP431142 |
| *Anisakis simplex s.l.* (NZ3) | Scarlett wrasse (Fish3Cnem1_17) | OP503278 |  |  |  | OP431143 |
| *Anisakis simplex s.l.* (NZ4) | Arrow squid (NOS2nem1_14) | OP503279 |  |  |  | OP431144 |
| *Anisakis simplex s.l.* (NZ5) | Salvin’s albatross (SA3nem1) | OP503280 |  |  |  | OP431145 |
| *Anisakis simplex s.l.* (NZ6) | Mullet (Mull2nem1_39) | OP503281 |  |  |  | OP431146 |
| *Anisakis simplex s.l.* (NZ7) | Silver warehou (SWH2nem1_24) | OP503282 |  |  |  | OP431147 |
| *Anisakis simplex s.l.* (NZ8) | Scarlett wrasse (SWR1nem1_24) | OP503283 |  |  |  | OP431148 |
| *Anisakis simplex s.l.* (NZ9) | Blue cod (BCD1nem1_38) | OP503284 |  |  |  | OP431149 |
| *Anisakis simplex s.l.* (NZ10) | Tarahiki (SWR3nem1_18) | OP503285 |  |  |  | OP431150 |
| *Anisakis simplex s.l.* (NZ11) | White-chinned petrel (WCPGnem1) | OP503286 |  |  |  | OP431151 |
| *Anisakis simplex s.l.* (NZ12) | Northern giant petrel (NGP2nem1) | OP503287 |  |  |  | OP431152 |
| *Anisakis simplex s.l.* (NZ13) | Olive rockfish (ORF1nem1_14) | OP503288 |  |  |  | OP431153 |
| *Anisakis simplex s.l.* (NZ14) | Sprat sp. 2 (SPM2nem1_41) | OP503289 |  |  |  | OP431154 |
| *Anisakis simplex s.l.* (NZ15) | Banded wrasse (Fish4Anem1_17) | OP503290 |  |  |  | OP431155 |
| *Anisakis simplex s.l.* (NZ16) | Mullet (Mull1nem1_10) | OP503291 |  |  |  | OP431156 |
| *Anisakis simplex s.l.* (NZ17) | Pigfish (PIGCnem1_41) | OP503292 |  |  |  | OP431157 |
| *Anisakis simplex s.l.* (NZ18) | White-capped mollymawk (SMO1nem2) | OP503293 |  |  |  | OP431158 |
| *Anisakis simplex s.l.* (NZ19) | Barracouta (BAR1nem2_17) | OP503294 |  |  |  | OP431159 |
| *Anisakis simplex s.l.* (NZ20) | White-chinned petrel (Petrel2_10_nem1) | OP503295 |  |  |  | OP431160 |
| *Anisakis simplex s.l.* (NZ21) | Scarlett wrasse (SWR1nem1_38) | OP503296 |  |  |  | OP431161 |
| *Anisakis simplex s.l.* (NZ22) | Thornfish (TRP1nem1_18) | OP503297 |  |  |  | OP431162 |
| *Anisakis* sp. 2 (NZ23) | White-chinned petrel (SB36nem2) | OP503298 |  |  |  | OP431163 |
| *Anisakis* sp. indet. | Variable oystercatcher (VOC2Jnem1) | OP503299 |  |  |  |  |
|  | Westland petrel (WLP9nem1) | OP503300 |  |  |  |  |
|  | Sooty shearwater (MB12nem1) | OP503301 |  |  |  |  |
|  | King shag (BLU3nem) | OP503302 |  |  |  |  |
|  | Flesh-footed shearwater (FFS1nem1) | OP503303 |  |  |  |  |
|  | Grey petrel (GPTnem1) | OP503304 |  |  |  |  |
|  | Black petrel (BPTnem1) | OP503305 |  |  |  |  |
|  | Common diving petrel (CDPnem1) | OP503306 |  |  |  |  |
|  | Triplefin spp. (TF1nem1) | OP503307 |  |  |  |  |
|  | Common roughy (CRO1nem1) | OP503308 |  |  |  |  |
|  | Red gurnard (GURnem1) | OP503309 |  |  |  |  |
|  | Blue warehou (WAR3nem1) | OP503310 |  |  |  |  |
| *Contracaecum rudolpii* E (NZ1) | Yellow-eyed penguin (YEP1nem1) | OP467567 |  | OP470848 | OP453432 |  |
|  | Little blue penguin (LBP6nem1) | OP467568 |  | OP470849 | OP453433 |  |
|  | Fiordland crested penguin (FCP2nem2cont) | OP467569 |  | OP470850 | OP453434 |  |
|  | Caspian tern (Ctn1nem1upp) | OP467570 |  | OP470851 |  |  |
|  | Otago shag (sis20nem1) | OP467571 |  | OP470852 | OP453435 |  |
|  | Spotted shag (ssh4nem3) | OP467572 |  | OP470853 | OP453436 |  |
|  | Northern giant petrel (NGP3nem2) | OP467573 |  | OP470854 | OP453437 |  |
|  | Blue cod (BCD1nem2) | OP467574 |  | OP470855 | OP453438 |  |
|  | Sprat sp. 1 (SPR4nem2_28) | OP467575 |  | OP470856 | OP453439 |  |
|  | Red-billed gull (RGBAnemcon) | OP467576 |  |  |  |  |
| *Contracaecum rudolphii* E (NZ2) | King shag (BLU1nem1) | OP467577 |  | OP470857 | OP453440 |  |
| *Contracaecum rudolphii* (NZ3) | Leopard seal (LSLnem) | OP467578 |  | OP470858 | OP453441 |  |
| *Contracaecum rudolphii* (NZ4) | Northern royal albatross (NRA1nem1A) | OP467579 |  | OP470859 | OP453442 |  |
| *Capillaria* sp. | Southern pied oystercatcher (SIPO5nem1) | OP467580 |  |  |  |  |
| *Eucoleus* sp. | Red-billed gull (RGB2nem1) | OP467581 |  |  |  |  |
|  | Black-backed gull (BBG3nem1) | OP467582 |  |  |  |  |
